# Structures and Corresponding Functions of Five Types of Picornaviral 2A Proteins

**DOI:** 10.3389/fmicb.2017.01373

**Published:** 2017-07-21

**Authors:** Xiaoyao Yang, Anchun Cheng, Mingshu Wang, Renyong Jia, Kunfeng Sun, Kangcheng Pan, Qiao Yang, Ying Wu, Dekang Zhu, Shun Chen, Mafeng Liu, Xin-Xin Zhao, Xiaoyue Chen

**Affiliations:** ^1^Institute of Preventive Veterinary Medicine, Sichuan Agricultural University Chengdu, China; ^2^Key Laboratory of Animal Disease and Human Health of Sichuan Province, Sichuan Agricultural University Chengdu, China; ^3^Avian Disease Research Center, College of Veterinary Medicine, Sichuan Agricultural University Chengdu, China

**Keywords:** 2A protein, five types, non-conserved, structure, function, antiviral research

## Abstract

Among the few non-structural proteins encoded by the picornaviral genome, the 2A protein is particularly special, irrespective of structure or function. During the evolution of the *Picornaviridae* family, the 2A protein has been highly non-conserved. We believe that the 2A protein in this family can be classified into at least five distinct types according to previous studies. These five types are (A) chymotrypsin-like 2A, (B) *Parechovirus*-like 2A, (C) hepatitis-A-virus-like 2A, (D) *Aphthovirus*-like 2A, and (E) 2A sequence of the genus *Cardiovirus*. We carried out a phylogenetic analysis and found that there was almost no homology between each type. Subsequently, we aligned the sequences within each type and found that the functional motifs in each type are highly conserved. These different motifs perform different functions. Therefore, in this review, we introduce the structures and functions of these five types of 2As separately. Based on the structures and functions, we provide suggestions to combat picornaviruses. The complexity and diversity of the 2A protein has caused great difficulties in functional and antiviral research. In this review, researchers can find useful information on the 2A protein and thus conduct improved antiviral research.

## Introduction

**FIGURE 1:**
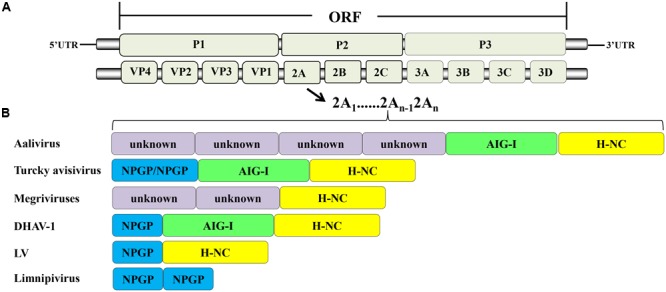
Picornavirus genome structure. **(A)** Protein processing of picornaviruses. **(B)** Some viruses have acquired multiple 2A genes, which occur in tandem in their genomes.

According to the ICTV, in March 2017, the family *Picornaviridae* consisted of 80 species grouped into 35 genera (Adams et al., 2016). The members of *Picornaviridae* are single-stranded, positive-sense RNA viruses. The genome contains a single ORF that encodes a precursor protein that includes the coding region of the capsid protein (P1) and non-structural proteins (P2 and P3). The precursor is cleaved into the mature proteins by viral proteins that act as proteases. The final structure is generally VP4-VP2-VP3-VP1-2A-2B-2C-3A-3B-3C-3D (**Figure 1A**). Most of the viruses in this family contain additional unique structural features in their genomes.

It has been reported that there are several types of non-structural 2A proteins. We believe that they can be divided into five types according to their structures and corresponding functions, with little correlation between each type. These types are (A) the chymotrypsin-like 2A, which contains a conserved catalytic triad, (B) the *Parechovirus*-like 2A, which has a conserved H-NC box, (C) the hepatitis-A-virus-like 2A, which is mainly involved in viral capsid assembly, (D) the *Aphthovirus*-like 2A, which, along with the first amino acid of 2B (P), has a “DxExNPGP” tail at its C-terminus (where ‘x’ is any amino acid), and (E) the 2A sequence of the genus *Cardiovirus*, which contains a DxExNPGP and a “YxxxxLΦ” (where Φ is a hydrophobic amino acid) motif. The highly complicated nature of 2A proteins has been an important factor hindering research. A comprehensive understanding of these proteins will greatly promote further research and clinical application of the research. In this paper, we focus on the existing reports on the five types of 2A to produce a relatively comprehensive review based on their structures and functions. We also provide suggestions for researchers to combat virus replication by targeting the 2A proteins.

## Evolutionary Analysis and Sequence Alignment of the Five Types of 2A Proteins

**FIGURE 2:**
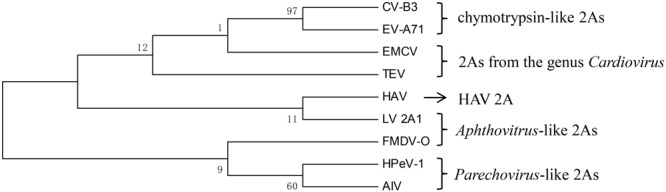
Phylogenetic tree based on the five types of 2A proteins. The tree was constructed using a ML method. The 2As of EV-A71 and CVB3 are chymotrypsin-like 2As. The 2As of HPeV-1 and AIV are *Parechovirus*-like 2As. The 2A of FMDV and the 2A1 of LV are *Aphthovirus*-like 2As. The 2As of EMCV and TEV are from the genus *Cardiovirus*. The numbers at the nodes represent the bootstrap value. (GenBank accession numbers: EV-A71, AAB39968.1; CVB3, AAB02228.1; HPeV-1, ABK54353.1; AIV, NP_740438.1; FMDV-O, ARB66056.1; LV, NP_705877.1; EMCV, NP_740406.1; TEV, NP_740428.1; HAV, ACD74580.1).

In addition to the hepatitis-A-virus-like 2A protein, two 2A proteins were selected from each type to build a phylogenetic tree (**Figure 2**). It can be seen that there are almost no similarities between each type. This suggests that there is no common evolutionary ancestor of the five types of 2A sequences. In addition, except for the chymotrypsin-like 2A proteins, the bootstrap value within each type is extremely low. Regarding the value for the chymotrypsin-like 2A proteins, this may be due to the fact that they function as a result of their overall conformation, so the full sequences are similar. In contrast, the other types of 2A function only due to several amino acids, and the remaining sequence is highly non-conserved. Therefore, we carried out a sequence alignment analysis separately for each type.

**FIGURE 3:**
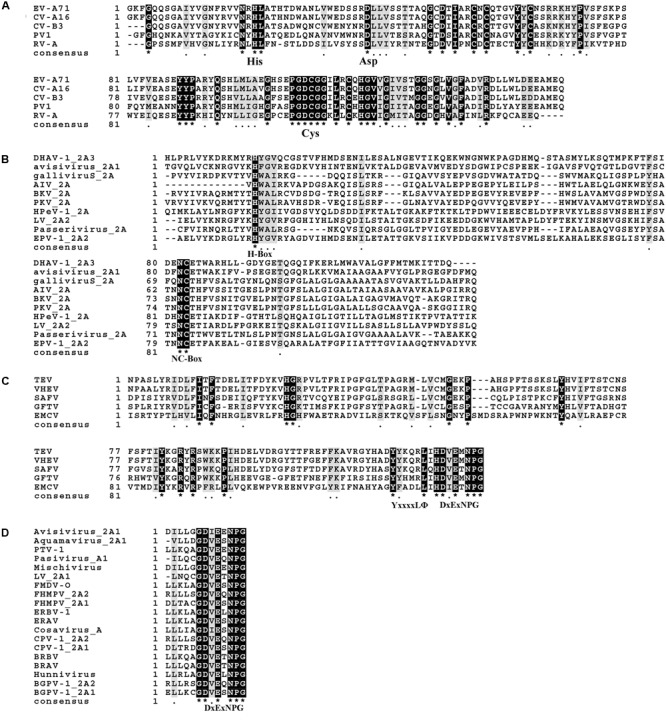
Sequence alignment of each type of 2A protein. The alignment was completed using MEGA6 and ClustalX2. **(A)** Alignment of chymotrypsin-like 2As. **(B)** Alignment of *Parechovirus*-like 2As. **(C)** Alignment of 2As from the genus *Cardiovirus*. **(D)** Alignment of *Aphthovirus*-like 2As. The ^∗^ means that the amino acids at this site are identical.

It can be seen that the catalytic triad His-Asp-Cys, which represents the active site of chymotrypsin-like 2A, is highly conserved (**Figure 3A**). This chymotrypsin-like 2A, which functions as a proteolytic enzyme that cleaves the viral protein precursors and host proteins, can help the virus to evade host immune responses in multiple ways. Many researchers regard it as a key drug target, aiming at its active sites to combat the virus. The H-NC box of *Parechovirus*-like 2A is also highly conserved (**Figure 3B**). This H-NC box is involved in viral replication and host cell proliferation. The *Aphthovirus*-like 2A are all short peptides. The DxExNPGP motif appears to be highly conserved in the small number of amino acids that make up these peptides (**Figure 3D**). This motif induces a co-translation event known as ribosomal “skipping,” thus separating the viral protein at the “G↓P” site. Many basic scientific research studies and clinical experiments have used this type of 2A peptide as a tool to cleave the precursor protein. In the 2A sequences of the genus *Cardiovirus*, the DxExNPGP and YxxxxLΦ motifs are all conserved (**Figure 3C**). The DxExNPGP motif functions like *Aphthovirus*-like 2A. The YxxxxLΦ motif is a binding site for eukaryotic initiation factor 4E (eIF4E). No special functional motif has been found for the hepatitis-A-virus-like 2A (**Table 1**). Due to the distinct nature of each type of 2A protein, the subsequent sections of this review elucidate the structural motifs and functions of each type of 2A proteins in turn to help researchers to understand the 2A proteins of *Picornaviridae* more clearly.

**Table 1:** The five types of 2A proteins.

Types of 2A	Functional motif	Common functions performed
Chymotrypsin-like 2A	Cys-His-Asp	Affect host translation, apoptosis, interferon pathway, nuclear transport, induced cardiomyopathy
*Parechovirus*-like 2A	H-NC box	Viral replication and cell proliferation
Unique 2A in HAV	No relevant functional motif was identified	Involved in virus assembly
*Aphthovirus*-like 2A	DxExNPGP	Ribosomal skipping mechanism
Unique 2A sequence in cardiovirus	NPGP, eIF4E binding site, NLS	Ribosomal skipping mechanism,Affect host translation and nuclear localization

## Structure and Functions of Chymotrypsin-Like 2A Protease (2A^Pro^)

### Important Structural Features of Chymotrypsin-Like 2A^pro^

**FIGURE 4:**
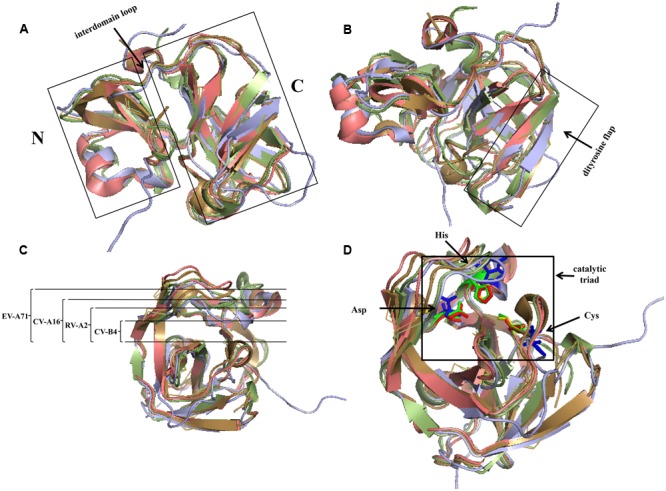
Comparison of structures of four common chymotrypsin-like 2A proteins using PyMOL. The 2A structures of RV-A2, CV-B4, CV-A16, and EV-A71 are represented in pale green, lilac, beige, and salmon, respectively. **(A)** The left and right domains are the N- and C-terminus, respectively, which are connected via an interdomain loop. **(B)** C-terminal dityrosine flap. **(C)** Open cleft across the surface of the protein. The width of EV-A71 is the largest, followed by that of CV-A16, and that of RV-A2 is the narrowest. **(D)** Active sites of 2A^pro^. The active sites of the 2As of RV-A2, CV-B4, CV-A16, and EV-A71 are represented in green, blue, yellow, and red, respectively. (PDB code: RV-A2, 2HRV; CV-B4, 1Z8R; CV-A16, 4MG3; EV-A71, 4FVD).

In members of *Picornaviridae*, most of the 2A proteins are chymotrypsin-related proteases. Comparing the structures of these 2A proteins, it can be shown that their overall conformations are similar (**Figure 4**). The typical structure of 2A^pro^ contains an N-domain comprising a four-stranded antiparallel β-sheet and a C-domain comprising a six-stranded antiparallel β-barrel; these domains are connected via a long interdomain loop (**Figure 4A**). Two β-strands form an antiparallel β-hairpin named the dityrosine flap within the C-domain (**Figure 4B**). There is an open cleft across the surface of the protein. The residues of the catalytic triad, Cys-His-Asp, are found in the cleft, which has conformational flexibility and thus fits well with the substrates (Cai et al., 2013). In different 2As, the different cleft widths adapt to the different substrates (**Figure 4C**). For example, 2A^pro^ of *Rhinovirus A* (RV-A) cannot process the same cleavage sites as 2A^pro^ of *Rhinovirus B* (Neubauer et al., 2013). Specific residues in 2A^pro^ can convert the cleft from a “closed” to an “open” state in a reversible manner (Sun et al., 2013). The catalytic site of this type of 2A^pro^ consists of the conserved catalytic triad (Cys-His-Asp). The catalytic site of poliovirus (PV) consists of Cys109-His20-Asp38 (Hellen et al., 1991). The active sites may be different in other viruses, but the overall conformation is maintained (**Table 2** and **Figure 4D**). Cys55, Cys57, Cys15, and His117 in PV 2A^pro^ are critical for maintaining its active conformation and catalytic activity (Yu and Lloyd, 1992). A zinc ion is required to maintain the conformation of 2A^pro^. However, when added externally, zinc ions appear to be inhibitory (Maghsoudi et al., 2008). The protease can be inhibited by a potent zinc chelator, but the protease will rapidly gain activity after the addition of excess zinc (Glaser et al., 2003). Despite these conserved features, some 2A^pro^ have distinctive properties. For example, the RV-C2 2A^pro^ has three short 3_10_-helices (Lee et al., 2014). A hydrophobic “LLWL” motif followed by an acidic “DEE” motif exists at the C-terminus of EV A71 (EV-A71) 2A^pro^ (Mu et al., 2013).

**Table 2:** Active sites of the catalytic triad in several chymotrypsin-like 2A proteins.

Active sites of catalytic triad	HRV-C2	HRV2	EV71	PV	CVB4	CVB3	CVA16
Cys	105	106	110	109	110	110	110
His	34	18	21	20	21	18	21
Asp	18	35	39	38	39	35	39

Several factors can affect the structure of 2A^pro^. Low temperature is necessary for the integrity of CV B3 (CV-B3) 2A^pro^ (Maghsoudi et al., 2008). Elevating the temperature will induce the active site to undergo a change (Maghsoudi et al., 2011). Structure determines function. The recognition of eIF4GI by CV-B4 2A^pro^ is driven by the unique surface properties (Baxter et al., 2006), and conformational change of the active site decreases the cleavage of eIF4G (Maghsoudi et al., 2011). The LLWL motif in EV-A71 2A^pro^ is critical for maintaining the conformation and positioning of the following acidic motif (DEE), the correct positioning of which is required for virus replication (Mu et al., 2013).

### Function of 2A^pro^ with Respect to the Virus Itself

Chymotrypsin-like 2A^pro^ cleaves the viral polyprotein between VP1 and 2A. When a special mutation was introduced between VP1 and 2A in RV-A1, VP1/2A could not be cleaved by 2A^pro^. The C-terminal three residues of VP1 are necessary for this cleavage in CV-B1 (Muto et al., 2006). It has shown that 2A^pro^ can cleave foreign proteins inserted between VP1 and 2A in an attenuated but infectious and replicative CV-B3 genome (Zeng et al., 2013). PV 2A^pro^ can cleave the polyprotein accurately after a red fluorescent protein (DsRed) is inserted after residue 144 in 2A (Teterina et al., 2010). This indicates that the *cis*-cleavage function can be used to develop expression vectors.

In addition to its *cis*-cleavage function, 2A^pro^ can also influence virus replication. PV 2A^pro^ is required for viral polysome formation and stability (Kempf and Barton, 2008). PV 2A^pro^, together with the 5′ cloverleaf-poly(C) binding protein complex and 3′ poly(A) tail, is required for the optimal translation of PV RNA (Ogram et al., 2010). It is sufficient to induce eIF2-independent IRES-driven translation alone (Redondo et al., 2011). However, it is not a prerequisite for the replication of the PV genome (Igarashi et al., 2010). The 2A^pro^ of EV-A71 and CV-B3 exhibited strong transcriptional activities in yeast cells. The C-terminal acid domain is essential for the transcriptional activity of 2A^pro^ (Yang et al., 2010).

### Function of 2A^pro^ with Respect to the Host

**FIGURE 5:**
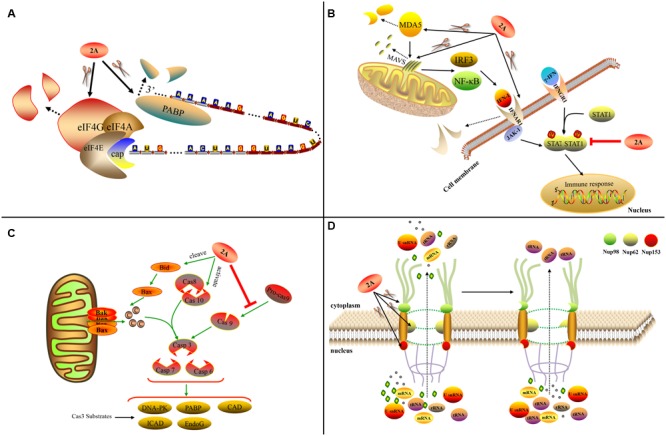
Functions of chymotrypsin-like 2A. **(A)** 2A^pro^ influences host translation. **(B)** 2A^pro^ influences the IFN pathway. **(C)** 2A^pro^ mediates apoptosis. **(D)** 2A^pro^ influences nucleo-cytoplasm transport (Sun et al., 2016).

During virus infection, 2A^pro^ plays a critical role in shutting down host protein synthesis (Lloyd, 2006). It cleaves eIF4G to halt cap-dependent host translation (Krausslich et al., 1987; Lloyd et al., 1988; Chau et al., 2007). The eIF4G protein is a subunit of eIF4F that facilitates mRNA unwinding and ribosome binding to mRNA. The eIF4F complex contains three subunits: eIF4E, eIF4G, and eIF4A. It has been shown that RV 2A^pro^ can cleave the eIF4G-eIF4E complex more efficiently than eIF4G alone (Haghighat et al., 1996). Moreover, 2A^pro^ from RV-A2 and CV-B4 can form a stable complex with eIF4GII/eIF4E, but not with eIF4GII alone (Aumayr et al., 2015). These findings suggest that the eIF4F complex may be a preferred substrate for this cleavage. RV 2A proteins cleave eIF4GI at different cleavage sites (Sousa et al., 2006). Cleaving only eIF4G does not completely shut down host translation. However, when 2A^pro^ also cleaves PABP, translational activity is completely prevented (Joachims et al., 1999) (**Figure 5A**). By cleaving eIF4G, PV 2A^pro^ can inhibit the synthesis of HIV-1 proteins, the translation of which is mainly cap-dependent during the early translational phase (Amorim et al., 2014). EV 2A^pro^-mediated eIF4G cleavage can also inhibit the IV IRES activity of duck hepatitis A virus (DHAV) (Pan et al., 2012). In contrast, this event can rescue the translation of Sindbis virus replicons when the genuine leader sequence of the mRNA is replaced by the picornaviral IRES (Sanz et al., 2010). CV-B3 2A^pro^ can also promote the replication of EMCV by inhibiting cellular cap-dependent translation (Song et al., 2015).

2A^pro^ plays an important role in apoptosis. PV 2A^pro^ can inhibit apoptosis during later-stage infection by inducing aberrant cleavage of procaspase-9 (Burgon et al., 2009). In contrast, CV-B3 2A^pro^ can induce apoptosis via multiple converging pathways. Firstly, CV-B3 2A^pro^ can induce caspase-8-mediated activation of caspase-3 and cleavage of PARP; secondly, it can activate the intrinsic mitochondria-mediated apoptosis pathway to induce the release of cytochrome *c* and activate caspase-9 via cleavage of BH3 interacting-domain death agonist (BID) (Chau et al., 2007) (**Figure 5C**).

Many picornaviruses target the IFN pathways to gain a replication advantage. The melanoma differentiation-associated gene 5 (MDA5)/mitochondrial antiviral-signaling protein (MAVS) pathway is responsible for recognizing EV infections (such as EV-A71 infection) of host cells and can induce IFN-I expression. Enteroviruses such as EV-A71 can employ 2A^pro^ to cleave MDA5 and MAVS, thus blocking the production of IFN-I (Feng et al., 2014). The cleavage sites in MAVS are Gly209, Gly251, and Gly265 (Wang et al., 2013). EV-A71 2A^pro^ can cleave the IFN receptor (IFN alpha and beta receptor subunit 1, IFNAR1) to block IFN-induced Janus kinase (Jak)/signal transducer and activator of transcription (STAT) signaling (Wang C.Y. et al., 2015). It can also cause the attenuation of IFN-γ signaling by reducing the serine phosphorylation of STAT1 (Wang L.C. et al., 2015) (**Figure 5B**). Viruses with chymotrypsin-like 2A^pro^ are able to replicate in IFN-α-pretreated cells while EMCV is completely inhibited; the addition of 2A^pro^ to the EMCV genome can prevent the inhibition. This suggests that 2A^pro^ plays an inhibitory role in the downstream event of IFN signaling (Morrison and Racaniello, 2009).

The traffic of biological molecules between the nucleus and cytoplasm occurs via the NPC, which is embedded in the nuclear membrane and is composed of multiple Nups. 2A^pro^ can influence the nucleo-cytoplasm by cleaving Nups, thus disrupting the structure of the NPC. PV 2A^pro^ cleaves Nup98, Nup62, and Nup153 to interfere with the trafficking of mRNAs, rRNAs, and U small nuclear RNAs (U snRNAs) from the nucleus to the cytoplasm, but without affecting the transport of tRNA (Castello et al., 2009) (**Figure 5D**). RV-A2 2A^pro^ cleaves Nup62 at six sites (Park et al., 2010) and Nup98 at two key sites, Gly374 and Gly552 (Park et al., 2015). Different RV 2A proteins cleave Nups at different sites (Watters and Palmenberg, 2011). In addition, 2A^pro^ can affect the location of several specific biomolecules. PV and RV-A16 2A^pro^ can induce efficient cytoplasmic relocation of the cellular splicing factor SRp20, which is an important IRES trans-acting factor for PV IRES-mediated translation (Fitzgerald et al., 2013). PV 2A^pro^ also induces the selective nucleo-cytoplasm translocation of HuR and TIA1/TIAR in order to modulate splicing of the human Fas exon 6 (Alvarez et al., 2013). In addition, PV 2A^pro^ can cause the viral proteins 3CD and 3C’ to move to the nucleus from the cytoplasm (Tian et al., 2011).

CV-B3 2A^pro^ can cleave the cytoskeletal protein dystrophin, thus causing severe dilated cardiomyopathy in the host (Xiong et al., 2007; Lim et al., 2013). Moreover, the release of the C-terminal fragment of dystrophin can cause more severe cardiomyopathy (Barnabei et al., 2015). CV-B3 2A^pro^ can also cleave serum response factor (Wong et al., 2012) and interact with human cardiomyocyte proteins (Zhao et al., 2016) to induce dilated cardiomyopathy, but the detailed mechanisms are unclear.

In addition to the common functions above, different 2A proteins also have several other functions to resist the host response. EV-A71 2A^pro^ can cleave the spliced X-box-binding protein 1 that is required for plasma cell differentiation (Jheng et al., 2012). It can also cleave the NACHT, LRR, and PYD domains-containing protein 3 (NLRP3) inflammasome at the G493-L494 junction to resist the host immune response during later-stage infection (Wang H. et al., 2015). CV-B3 2A^pro^ can downregulate the expression of cyclic AMP responsive element binding protein, which functions as a transcription factor (Chau et al., 2007), and cleave the Grb2-associated binder 1, which is an important docking protein responsible for intracellular signaling system assembly and signal transduction (Deng et al., 2015). CV-B3 and EV-A71 2As can trigger stress granule formation (Wu et al., 2014). RV 2A^pro^ can activate monocyte-derived dendritic cells *in vitro* and induce strong Th1 and Th2 immune responses from CD4 T cells during upper and lower respiratory tract infections in patients with severe chronic obstructive pulmonary disease (Singh et al., 2010).

### Antiviral Inhibitors of 2A^pro^

In summary, 2A^pro^ plays an essential role in resisting host defenses during virus infection. It evades the host immune responses in multiple ways. Blocking the interaction of the virus with the host can effectively improve host resistance, thus increasing resistance against virus infection. Due to its unique protein structure and its essential role in viral replication, 2A^pro^ is regarded as an excellent target for antiviral intervention. A great number of inhibitors have successfully been developed as substrate analogs that bind to the protein’s active sites (Wang, 2001). The short peptide LVLQTM and a modified form, z-LVLQTM-fmk, can both specifically inhibit 2A^pro^ (Falah et al., 2012a,b). The novel compound CW-33 can inhibit the enzymatic activity of 2A^pro^, thus recovering the expression of IFNAR1 in EV-A71-infected cells (Wang C.Y. et al., 2015). A synthesized 16-mer peptide can also specifically block the activity of CV-B3 2A^pro^ (Maghsoudi et al., 2010). In addition, several drugs can inhibit the activity of 2A^pro^ via other mechanisms. The indirubin derivative E804 inhibits the 2A^pro^-dependent cleavage of 3CD, thus interfering with CV-B3 polyprotein processing (Ford Siltz et al., 2014). zVAD.fmk is a general inhibitor and its derivative zVAM.fmk can specially inhibit RV 2A^pro^ (Deszcz et al., 2006). Chlorogenic acid, which is the major active ingredient in many traditional Chinese herbs, exhibits antiviral properties against EV-A71 by inhibiting transcription and translation of 2A^pro^ (Li et al., 2013).

## Conserved Motifs and Functions of *Parechovirus*-Like 2A

The *Parechovirus*-like 2A sequences are not proteases. This type of 2A has a conserved H-NC box, and some versions have a putative transmembrane domain (Hughes and Stanway, 2000). These 2A proteins are related to the cellular H-rev107 family, the members of which are involved in the control of cell proliferation (Tsai et al., 2009; Wei et al., 2015). In addition, the H-NC box is involved in virus replication. During HPeV infection, the bulk of the 2A proteins localize to the perinuclear area and some colocalize with the viral RNA (Samuilova et al., 2004; Krogerus et al., 2007). *Parechovirus*-like 2A possesses RNA-binding activity and preferentially binds to the viral 3′ untranslated region (UTR) (Samuilova et al., 2004). *Parechovirus*-like 2A can interact with all of the non-structural proteins except for L and 3AB. The strongest interaction occurs between 2A and 3CD (Ishikawa et al., 2010). In AIV, the efficient cleavage of VP1/2A requires interaction between 3CD and 2A (Sasaki et al., 2012). The H-NC box in the 2A protein is essential for AIV replication. Mutation of the NC motif abolishes virus replication by affecting both negative- and positive-strand synthesis (Sasaki and Taniguchi, 2008). These findings indicate that this type of 2A may play important roles in replication complexes by interaction with viral proteins and RNA during viral RNA replication. Targeting this 2A protein may affect the formation of the replication complex, thus combatting virus proliferation.

## Unique 2A Protein in Hepatitis A Virus (HAV)

**FIGURE 6:**
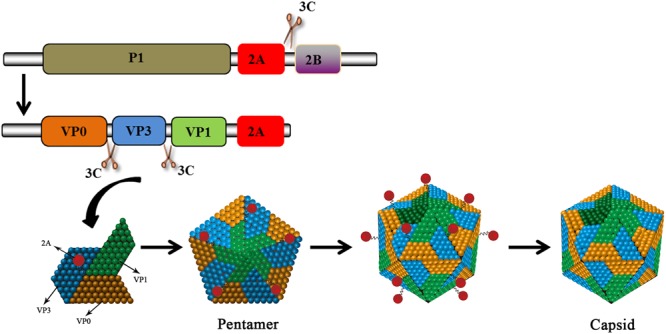
HAV 2A is involved in viral capsid assembly. The HAV protein precursor is cleaved by 3C^pro^ to produce VP0, VP3, and VP1-2A. The products assemble into pentamers that then assemble into icosahedral viral capsids. 2A (as part of VP1-2A) is involved in virus assembly. The VP1/2A junction is then cleaved by cellular proteases to release 2A.

No functional motifs have been found in the 2A gene of HAV. This type of 2A protein is considered a structural component and it is involved in morphogenesis. The HAV 2A protein, together with the VP1 protein as a precursor, is responsible for the virulence of HAV (Emerson et al., 2002). The deletion of 10–15aa from the central portion of the 2A protein does not affect the protein’s processing and the mutant virus is able to replicate relatively well (Harmon et al., 1995). The 2A protein does not function *in cis* as a 2AB precursor (Beard et al., 2001). The cleavage at the HAV 2A/2B junction is carried out by 3C^pro^ at Gln836/Ala837 (Jia et al., 1993; Martin et al., 1995). The released P1-2A acts as a functional precursor. 2A, as a part of P1-2A, is a primary signal for the assembly of pentameric viral structures (Probst et al., 1998, 1999). 3C^pro^ subsequently cleaves the precursor to generate VP0, VP3, and VP1-2A. These products, which associate as pentamers, constitute the icosahedral viral capsid. Subsequently, cleavage occurs at the VP1/2A junction to release the mature 2A protein (Cohen et al., 2002). This cleavage is carried out by cellular proteinases such as Factor Xa, trypsin, and cathepsin L (**Figure 6**) and it requires Arg^278^ to be present at the VP1/2A junction (Rachow et al., 2003; Morace et al., 2008). Targeting this 2A protein may interfere the formation of the icosahedral viral capsid. In addition, the 2A protein can strongly inhibit human growth hormone (the translation of which is entirely cap-dependent) via an unknown mechanism (Maltese et al., 2000). VP1-2A can also reduce the viability of Huh-7 cells (Kanda et al., 2006).

## Mechanism and Function Mediated By *Aphthovirus*-Like 2A Peptide

### Ribosomal Skipping Mechanism for “Self-Cleavage” in the Viral Translation Process

**FIGURE 7:**
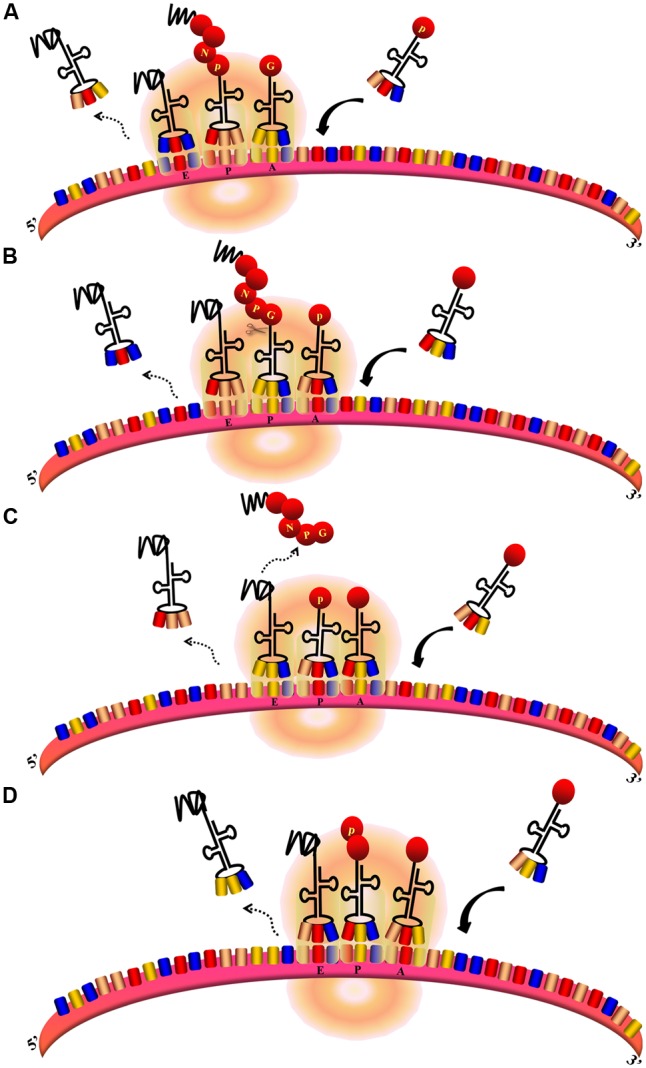
Ribosomal skipping mechanism mediated by *Aphthovirus*-like 2A sequences (Donnelly et al., 2001b). **(A,B)** During the peptide chain extension process, the ribosome moves toward the 3′UTR of the mRNA. The deaminoacyl-tRNA at the E site is released, the peptidyl–tRNA at the P site is transferred to the E site, and the aminoacyl-tRNA at the A site is transferred to the P site to allow the next aminoacyl-tRNA to enter the A site. At the same time, the peptide chain at the E site is transferred to the P site and forms a peptide bond with the amino acid at this site. **(B–D)** When the translation of 2A is finished, aminoacyl-tRNA^(Pro)^ enters the A site. The peptide chain is hydrolyzed from the peptidyl–tRNA at the P site. The depeptidyl-tRNA is then transferred to the E site and the aminoacyl-tRNA^(Pro)^ is transferred to the P site. The new aminoacyl-tRNA enters the A site as normal. Subsequent translation proceeds normally.

The FMDV 2A protein (F2A) in *Aphthovirus* is a short peptide containing only 18aa. 3C^pro^ can only successfully process the P1 polyprotein into the structural protein VP1 when 2A is present (Guo et al., 2013). However, the 2A protein does not affect the assembly of empty capsid particles nor the viability of the virus (Gullberg et al., 2013). Along with the first residue of 2B, the 2A sequence comprises a conserved C-terminal DxExNPGP motif. This 2A-like sequence is found in many mammalian viruses and a wide range of insect viruses (Luke et al., 2008). The DxExNPGP motif induces a co-translational “cleavage” event that releases P1-2A from the P2 polyprotein. This permits the translation of P2 after P1-2A is released from the ribosome (Donnelly et al., 2001b; Grubman and Baxt, 2004). The cleavage requires neither eIFs nor eRFs. The cleavage is carried out by the ribosome and eukaryotic elongation factors 1 and 2 (eEF-1 and -2) (Machida et al., 2014). It is assumed that F2A promotes hydrolysis of the peptidyl (2A)-tRNA^Gly^ ester linkage, thus releasing the polypeptide from the translational complex. This process causes the ribosome to “skip” from one codon to the next without forming a peptide bond (**Figure 7**). The ribosomal skipping activity can be affected by the C-terminal sequence of the protein upstream. The 30–40aa upstream sequence highly influences the skipping activity (Minskaia and Ryan, 2013). The upstream capsid VP1 sequences increase the skipping activity of F2A. N-terminal truncation of 2A sequences decreases the skipping activity and 13 residues are required for minimum activity. The “uncleaved” polyprotein cannot be cleaved later (Ryan and Drew, 1994). When using artificial polyprotein systems, there was a molar excess of the N-terminal product of this translational event compared with the C-terminal product; however, this phenomenon does not occur in the natural P12A2B2C context (Donnelly et al., 2001b). This is consistent with the idea that the artificial systems do not represent the optimal functioning environment for this translational event; not all ribosomes continue to synthesize the protein downstream (Donnelly et al., 2001a). The mutation of D within the DxExNPGP motif abrogates the F2A cleavage activity (Donnelly et al., 1997). The synonymous mutation of G has no effect on the skipping efficiency (Gao et al., 2014). In contrast, mutation of the first G to D in the GxExNPGP motif of the 2A protein of infectious flacherie virus abolishes the skipping activity (Donnelly et al., 2001a). F2A does not exhibit the skipping activity in prokaryotic translation systems (Donnelly et al., 1997).

Simultaneous expression of multiple genes is frequently required in genetic engineering (de Felipe et al., 1999). The *Aphthovirus*-like 2A sequences are increasingly preferred for this purpose due to their small size and high cleavage efficiency (de Felipe, 2004). Compared with IRES, 2A mediates a more efficient expression of the downstream protein (Chan et al., 2011), with levels up to ∼4 times greater than those mediated by IRES (Chinnasamy et al., 2006). It has been shown that the incorporation of viral 2A sequences into polycistronic vectors does not cause unwanted immune responses against TD cells (Arber et al., 2013). For biomedical applications, the 30 residues of F2A have been shown to be the most optimal residues in terms of both length and cleavage efficiency (Minskaia et al., 2013). The cleavage efficiencies of 2A sequences from FMDV, ERAV, TAV, and PTV-1 show that the 2A protein of PTV-1 has the highest cleavage efficiency (Kim et al., 2011). *Aphthovirus*-like 2A has been used to express transgenes in multiple circumstances. It can be used in oncolytic adenoviruses (Funston et al., 2008), lentiviral vectors (Chinnasamy et al., 2006), retroviral vectors (de Felipe et al., 1999), recombinant adeno-associated viruses (Furler et al., 2001), and influenza virus vectors (Tan et al., 2016) to express multicistronic genes.

### Application of *Aphthovirus*-Like 2A

The *Aphthovirus*-like 2A has been successfully used in multiple expression systems, including those in plants and parasites, demonstrating its extensive application range. It has been used to efficiently co-express the two functional subunits of bovine interleukin-12 (IL-12) (Chaplin et al., 1999), subunit p19 of IL-23, p40 of IL-12 (Yen and Scheerlinck, 2013), and the heavy and light chains of full-length coagulation factor VIII (He et al., 2011). The co-expression of porcine IFN-α and IFN-γ using F2A can enhance their antiviral effects against FMDV (Kim et al., 2014). F2A significantly improved the lentiviral Cre recombinase delivery system designed to provide a specific Cre activity level (Liu et al., 2012).

This 2A can also ensure stable expression of multiple proteins introduced into plant cells (Halpin et al., 1999). It does not affect the localization of the co-expressed proteins, so it is a useful tool for studying plant intracellular trafficking (Buren et al., 2012). The co-expression of lignocellulose hydrolysis enzymes linked by the F2A protein provides a novel low-cost enzyme system for hydrolyzing lignocellulosic material (Lee et al., 2012). In microalgae, F2A can be used to resolve a major obstacle of poor expression of heterologous genes in the nuclear genome (Rasala et al., 2012). In addition, F2A has also been used to co-express glycine betaine synthesis genes in the yeast *Pichia pastoris* (Wang et al., 2007). Moreover, the F2A-mediated heterogeneous glycosylphosphatidylinositol-anchored proteins co-displaying yeast system is an effective method for improving the efficiency of enzyme-displaying yeast biocatalysts (Sun et al., 2012). In addition, F2A has been used to co-express five *Taenia solium* (pork tapeworm) immunogens in plant cells (Monreal-Escalante et al., 2015). The 2A protein of PTV-1 has also been successfully used to co-express multi-proteins in parasites (Tang et al., 2016).

## Conservative Motif of Function of the Unique 2A Sequence In *Cardiovirus*

The members of the genus *Cardiovirus* possess a unique 2A protein. This protein has a DxExNPGP motif, an YxxxxLΦ motif, and a NLS. The ribosomal skipping motif functions effectively (Donnelly et al., 2001a). The YxxxxLΦ motif fits the eIF4E binding site used by eIF4G to interact with eIF4E (Groppo et al., 2011). The L proteins of EMCV, TMEV, and SAFV use the NLS in 2A to enhance their trafficking to the nucleus; in turn, 2A prevents L phosphorylation (Petty et al., 2014). Binding to host eIF4E is a potential mechanism by which the 2A protein shuts down host translation (Groppo et al., 2011). In addition, EMCV 2A can shut down host translation by tightly associating with free 40S ribosome subunits (Groppo and Palmenberg, 2007). The 2A protein is also required for inhibition of apoptosis (Carocci et al., 2011), probably because apoptosis can affect the survival of the virus. In addition, during EMCV infection, recombinant adenovirus containing the precursor protein P1/2A and 3C^pro^ can elicit more efficacious protection than the one containing P1 alone (Chen et al., 2008). This indicates the necessity of processing P1/2A to generate a sufficient neutralizing antibody response.

## Viruses Containing Multiple 2A Proteins

Most of the members of *Picornaviridae* have only one 2A protein, but during evolution, some viruses miraculously acquired multiple 2As. These multiple 2A proteins in single viruses can be the same or different, and can thus perform corresponding or divergent functions. This phenomenon is unique to picornaviruses. The members of *Limnipivirus* contains two *Aphthovirus*-like 2A proteins (Barbknecht et al., 2014; Lange et al., 2014). The LV also contains two 2A proteins, but one is *Aphthovirus*-like 2A and the other is *Parechovirus*-like 2A (Johansson et al., 2002). It has been reported that DHAV-1 has three in-tandem 2A genes (Tseng and Tsai, 2007b; Jin et al., 2008). They encode the 2A1, 2A2, and 2A3 proteins, which are *Aphthovirus*-like 2A, AIG1-like 2A with a GTPase motif, and *Parechovirus*-like 2A, respectively (Tseng and Tsai, 2007a; Tseng et al., 2007). In contrast, other researchers suggest that DHAV-1 contains only two 2A proteins, an *Aphthovirus*-like 2A1 and a *Parechovirus*-like 2A2 with an AIG1 domain at the N-terminus (Kim et al., 2006; Ding and Zhang, 2007). Viruses in the genus *Megrivirus* possess three potential 2A peptides, one of which, 2A3, contains an H-NC box (Boros et al., 2014b; Liao et al., 2014). A turkey avisivirus 1 (TuASV-1) possesses three 2A proteins (potentially similar to DHAV-1), but the 2A1 protein contains two DxExNPGP motifs (Boros et al., 2013). The newly discovered picornavirus Aalivirus contains six 2A proteins, including four *Aphthovirus*-like 2As (2A1–2A4), an AIG1-like 2A5, and a *Parechovirus*-like 2A6 (Wang et al., 2014) (**Figure 1B**). The diversity of 2A proteins suggests that multiple recombination events occurred during their evolution. It appears that the 2A proteins have provided a very strong advantage to picornaviruses during evolution in that they provide multiple powerful functions for use during viral infection. The viruses that have acquired multiple 2A proteins have mostly been obtained from avian hosts (Boros et al., 2014c), suggesting that picornaviruses infecting avian hosts may have more complex mechanisms than other picornaviruses.

## Conclusion

Among the five types of 2A proteins, chymotrypsin-like 2A^pro^ represents a relatively large category. For 2A^pro^, substrate recognition does not involve the certain amino acid sequences but rather undetermined restricted spatial factors (Neubauer et al., 2013), so its conformation is important for maintaining its function. 2A^pro^ prevents host immune responses via multiple mechanisms and utilizes host factors to enhance viral replication. The development of inhibitors of 2A^pro^ requires a rational strategy based on its structure and function. We can improve upon the existing inhibitors that target the structure of 2A^pro^ to improve resistance to viral infection.

The *Aphthovirus*-like 2A sequence that mediates the ribosomal skipping mechanism represents another large category of 2A proteins. These short 2A peptides can mediate a unique translation mechanism alone. It is still unclear what factors are involved in the translation process; although a tentative functional mechanism has been suggested, it needs to be explored further. These short 2A peptides have been widely used in genetic engineering to co-express multiple genes. Compared to other co-expression methods, this technique offers powerful advantages. Nevertheless, it still has several flaws that have yet to be overcome. Solving these problems will help to improve the practical application of the short 2A peptides.

In addition to the two major classes of 2A proteins, all other 2A proteins play important roles in virus replication and resisting host immune responses via distinctive mechanisms. These complicated functions and corresponding structures still require more in-depth exploration.

The evolution of the 2A protein has been extremely fast, and diverse 2A proteins have evolved. The highly non-conserved nature of the 2A protein causes great difficulty for researchers. Its unusually rapid evolutionary rate suggests that it is involved in additional host interactions (Hughes, 2004). This indicates that 2A plays a pivotal role in virus infection. It has been shown that the AIG-I-like 2A2 protein of DHAV-1 can induce apoptosis (Cao et al., 2016). As none of the five types of 2A protein reviewed in this paper possess this capacity, the AIG-I-like 2A2 protein of DHAV-1 may have to be classified as a distinct type of 2A protein. Moreover, there are some 2A proteins that have yet to be recognized (Boros et al., 2014a; Liao et al., 2014). The phenomenon of multiple types of 2A proteins existing in one virus family is extremely rare. In addition, although the phenomenon of multiple types of 2A proteins existing in one virus family is extremely rare, discovering why some viruses have acquired multiple 2As remains an important and challenging issue to be explored. All of the above problems cause great difficulties for researchers regarding using a single specific 2A protein as a model. The widespread prevalence of picornaviruses has caused a large number of cases of disease and large losses in production. Preventing these viral infections is an urgent problem to be solved. This review has provided evidence indicating the usefulness of targeting 2A when designing specific inhibitors and other strategies to resist picornaviruses.

## Author Contributions

XY wrote the manuscript and produced the figures. AC, MW, and RJ revised the manuscript. KS, KP, and QY provided the ideas for the figures. YW, DZ, and SC revised the figures. ML, X-XZ, and XC helped with proofreading. All of the authors edited the manuscript.

## Conflict of Interest Statement

The authors declare that the research was conducted in the absence of any commercial or financial relationships that could be construed as a potential conflict of interest.

## Abbreviations

AIV: Aichi virus
BGPV: Bluegill picornavirus
BKV: bovine kobuvirus
BRAV: bovine rhinitis A virus
BRBV: bovine rhinitis B virus
CPV: Carp picornavirus
CV: coxsackievirus
eEF: eukaryotic elongation factor
eIF: eukaryotic initiation factor
EMCV: encephalomyocarditis virus
EPV: Eel picornavirus
ERAV: equine rhinitis A virus
ERBV: equine rhinitis B virus
eRF: eukaryotic release factors
EV: enterovirus
FHMPV: fathead minnow picornavirus
FMDV: foot-and-mouth disease virus
F2A: foot-and-mouth disease virus 2A
GFTV: genet fecal theilovirus
HAV: Hepatitis A virus
HPeV: human parechovirus
ICTV: International Committee on Taxonomy of Viruses
IFN: interferon
IL: interleukin
IRES: internal ribosome entry site
LV: Ljungan virus
ML: maximum likelihood
NLS: nuclear localization sequence
NPC: nuclear pore complex
Nups: nucleoporins
ORF: open reading frame
PABP: poly (A)-binding protein
PKV: porcine kobuvirus
PTV-1: porcine teschovirus-1
PV: poliovirus
RV: rhinovirus
SAFV: saffold virus
TAV: thosea asigna virus
TEV: theilovirus
TMEV: Theiler’s murine encephalomyelitis virus
VHEV: Vilyuisk human encephalomyelitis virus
2A^pro^: 2A protease
